# Supplementary figures and images for: Salorno—Dos de la Forca (Adige Valley, Northern Italy): A unique cremation site of the Late Bronze Age
Source: PLoS One. 2022 May 18;17(5):e0267532. doi: 10.1371/journal.pone.0267532 (PMC9116657; doi:10.1371/journal.pone.0267532)

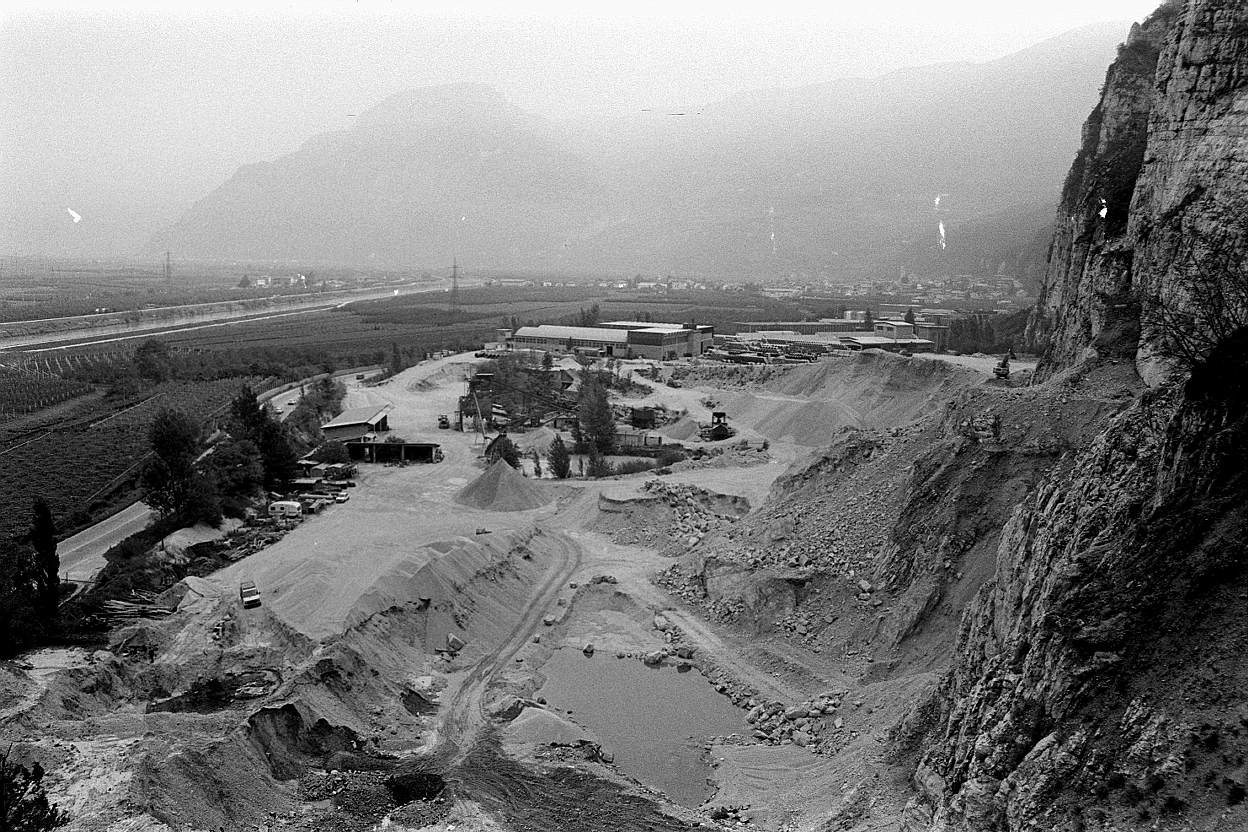

Supplement: S1 Fig — (TIF) [file pone.0267532.s001.tif]

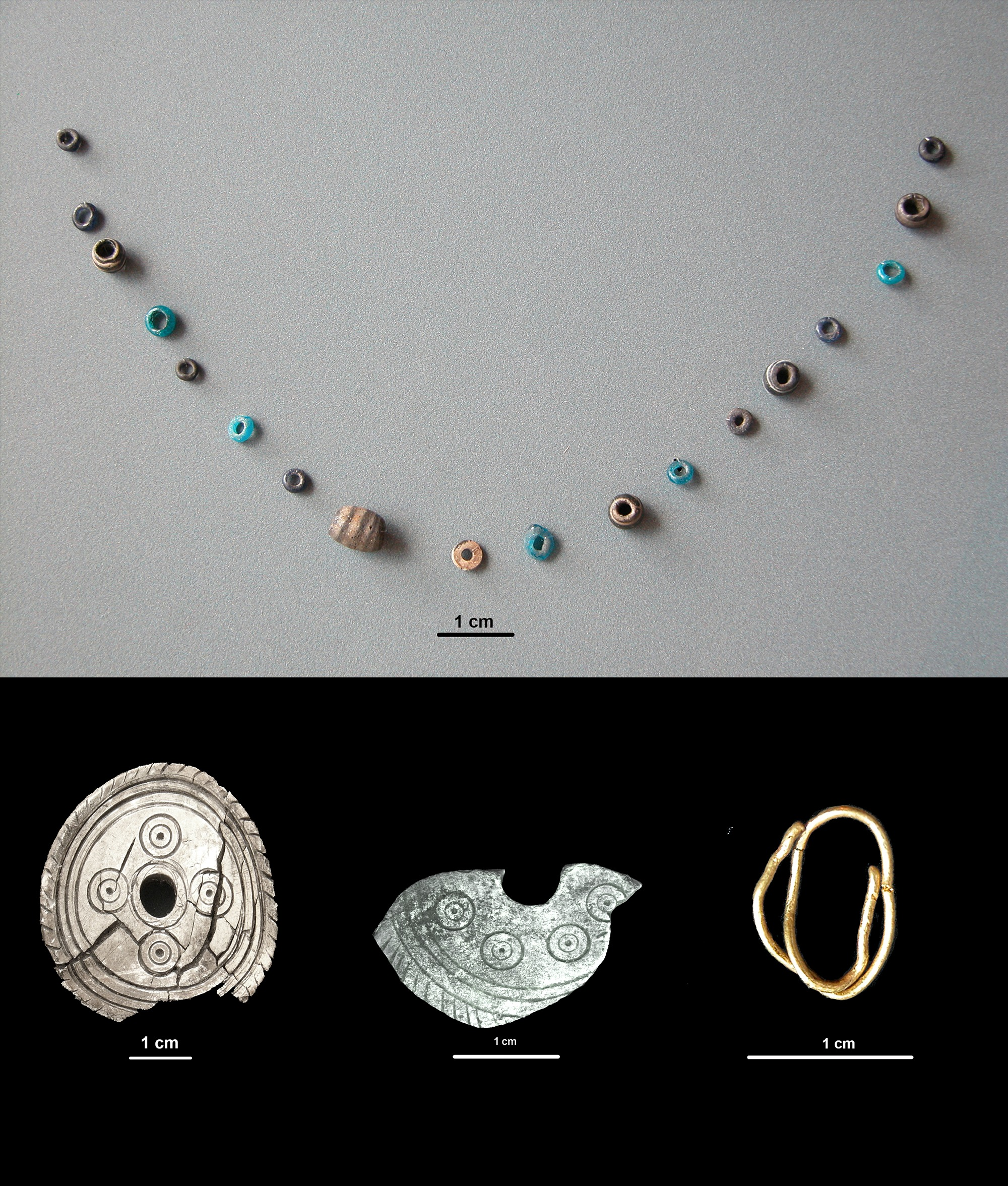

Supplement: S2 Fig — (TIF) [file pone.0267532.s002.tif]

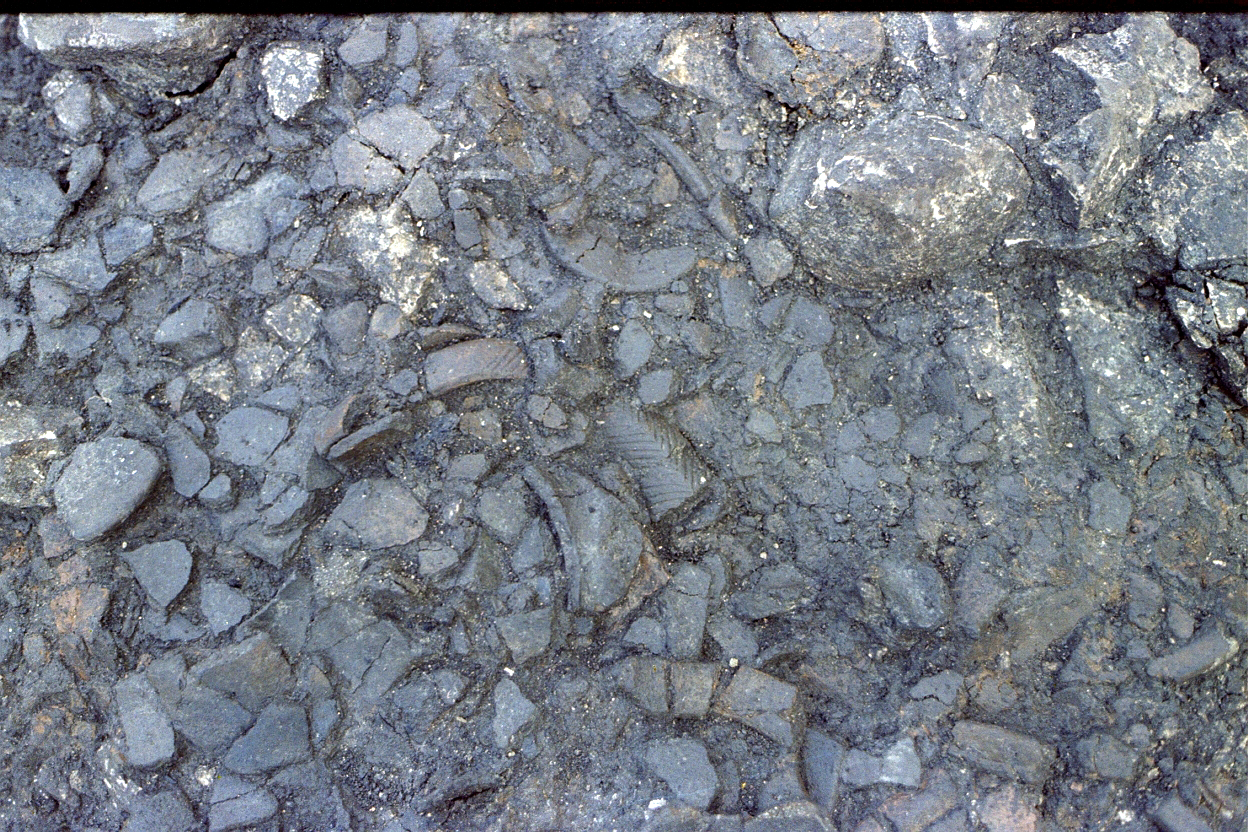

Supplement: S3 Fig — (TIF) [file pone.0267532.s003.tif]

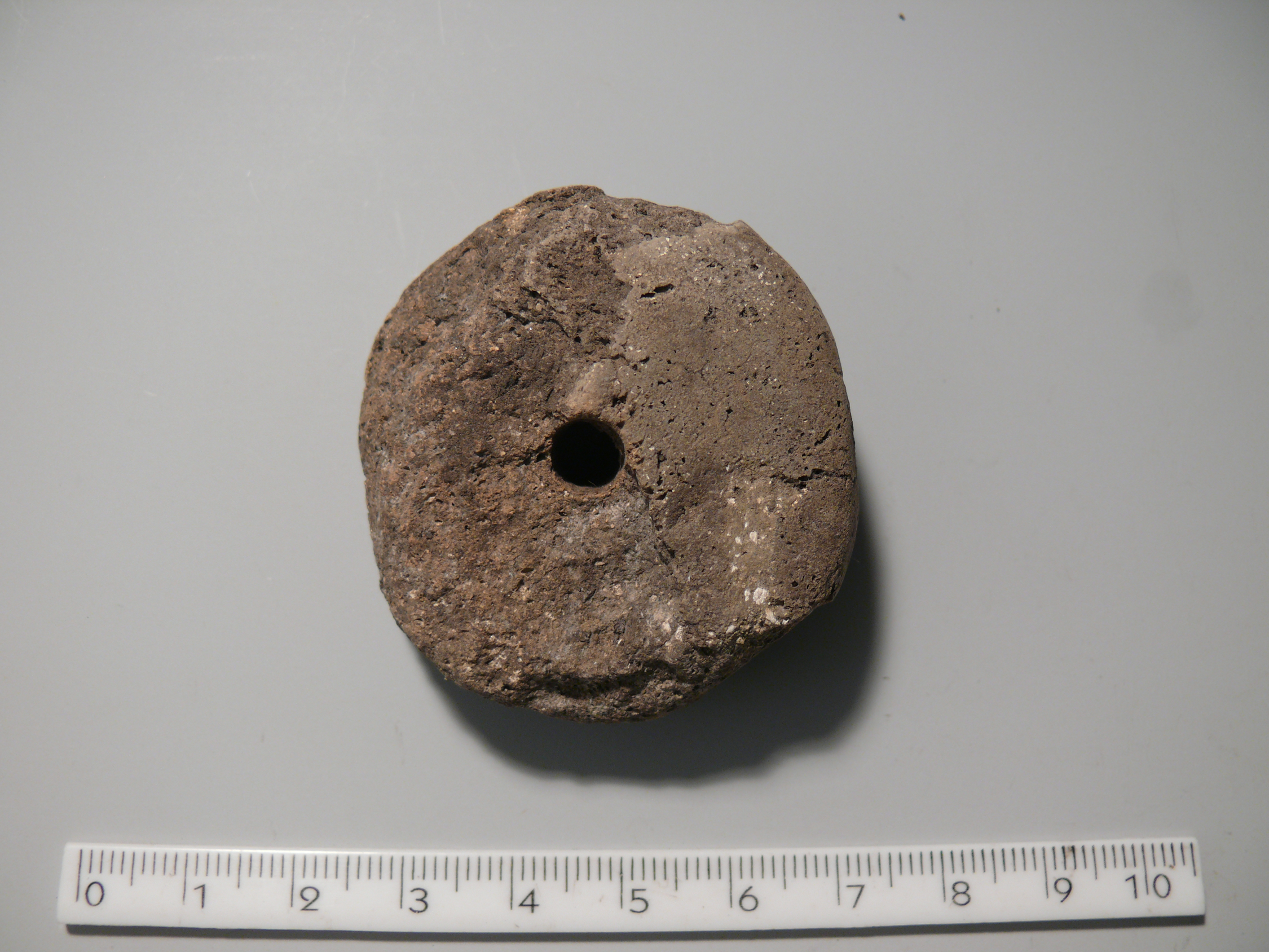

Supplement: S4 Fig — (TIF) [file pone.0267532.s004.tif]
